# Screening and identification of DNA aptamers toward *Schistosoma japonicum* eggs via SELEX

**DOI:** 10.1038/srep24986

**Published:** 2016-04-28

**Authors:** Yuqian Long, Zhiqiang Qin, Minlan Duan, Shizhu Li, Xiaoqiu Wu, Wei Lin, Jianglin Li, Zilong Zhao, Jing Liu, Dehui Xiong, Yi Huang, Xiaoxiao Hu, Chao Yang, Mao Ye, Weihong Tan

**Affiliations:** 1 Molecular Science and Biomedicine Laboratory, State Key Laboratory for Chemo/Biosensing and Chemometrics, College of Biology, College of Chemistry and Chemical Engineering, Collaborative Innovation Center for Molecular Engineering for Theranostics, Hunan University, Changsha, Hunan 410082, China; 2 National Institute of Parasitic Diseases, Chinese Center for Disease Control and Prevention, Shanghai 200025, China; 3 The State Key Laboratory of Medical Genetics and School of Life Sciences, Central South University, Changsha, Hunan 410078, China; 4 Hunan Provincial Center for Disease Control and Prevention, Changsha, Hunan 410005, China; 5 College of Life and Environmental Sciences, Gannan Normal University, Ganzhou, Jiangxi 341000, China; 6 Department of Chemistry, Department of Physiology and Functional Genomics, Center for Research at Bio/Nano Interface, University Health Cancer Center, University of Florida Genetics Institute and McKnight Brain Institute, University of Florida, Gainesville, FL, 32611-7200, USA.

## Abstract

Schistosomiasis is a major parasitic disease caused by blood flukes of the genus Schistosoma. Several million people all over the world are estimated to suffer from severe morbidity as a consequence of schistosomiasis. The worm’s eggs, which cause the symptoms of schistosomiasis, are generally used to diagnose the disease. In this study, we employed egg-based systematic evolution of ligands by exponential enrichment (egg-SELEX) and identified a panel of ssDNA aptamers specifically binding to eggs derived from *S. japonicum*. Among these, two aptamers LC6 and LC15 exhibited strong binding to and specific recognition of *S. japonicum* eggs, but not eggs from *Fasciolopsis buski*, *Enterobius*, *Ascaris* or *Clonorchis sinensis*. Furthermore, tissue imaging results revealed that LC15 could recognize *S. japonicum* eggs laid in liver tissues with a detection ratio of 80.5%. Collectively, therefore, we obtained useful aptamers specifically recognizing *S. japonicum* eggs, which will facilitate the development of an effective tool for both schistosomiasis diagnosis and drug delivery.

Schistosomiasis is a major parasitic disease caused by blood flukes of the genus Schistosoma. It affects the liver, mesentery, and urogenital tract of infected individuals. The World Health Organization (WHO) estimates that more than 200 million people are currently infected by this disease worldwide, resulting in a mortality rate exceeding 200,000 each year^1^. Most human schistosomiasis is caused by *Schistosoma haematobium* (*S. haematobium)*, *Schistosoma mansoni* (*S. mansoni* ) and *Schistosoma japonicum* (*S. japonicum*). Among these, *S. japonicum* is the major human blood fluke that occurs in Asia, particularly in China, the Philippines, Thailand, and Indonesia^2^. The ovulation rate of *S. japonicum* is approximately 2200 eggs daily per female adult worm. Schistosoma eggs can be deposited in the host organs, which causes the symptoms of schistosomiasis derived from host immune response to eggs trapped in tissues Egg-released antigens stimulate a granulomatous reaction involving T cells, macrophages, and eosinophils that results in clinical disease, such as granuloma and fibrosis^3^,^4^.

Schistosoma eggs can be emitted into water with the host feces, which is the beginning of schistosomiasis transmission. The current *in vitro* diagnostic method involves the demonstration of schistosome eggs in the stool, and it remains the gold standard for the diagnosis of schistosomiasis and the technique of choice for testing candidate drug efficacy. However, low-intensity infections reduce the sensitivity of other tests, such as the Kato-Katz thick smear method^5^. Although antibody-based detection techniques may be more sensitive, cross-reaction with other helminth infections often results in lower specificity, and these methods are also more sophisticated and expensive^6^,^7^.

Aptamers are short single-stranded oligonucleotide molecules (RNA or ssDNA) that undergo conformational changes that allow folding into unique tertiary structures able to bind specific cell-surface targets^8^. Similar to antibodies, aptamers possess high-binding affinity for their targets, typically from picomolar to nanomolar levels, depending on the nature of targets^9^. Comparable to antibodies, aptamers also recognize their targets with high specificity. For instance, aptamers can discriminate among homologous proteins that contain only a few amino acid changes^10^. In addition, aptamers exhibit some unique features over antibodies, including low molecular weight, high stability, easy and controllable synthesis and modification for different diagnostic and therapeutic purposes, lack of immunogenicity, rapid tissue penetration, and nontoxicity^11^. Owing to these molecular properties, aptamers have been widely employed as a novel tool for diagnosis and treatment of disease.

Aptamers can be generated from a synthetic random library with 10^13^–10^16^ ssDNA or ssRNA molecules by an *in vitro* iterative selection process called systematic evolution of ligands by exponential enrichment (SELEX)^12^,^13^. Over the last 20 years, aptamers have been successfully isolated for a variety of targets, ranging from small molecules, such as metal ions, organic dyes, amino acids, or short peptides, to large proteins or complex targets, including whole cells, viruses, bacteria, or tissues^14^,^15^,^16^. Especially, the selection against complex targets can be carried out without knowing the distinct molecular signature in individual target cells, suggesting that these molecular tools can support improved clinical diagnosis and treatment, as well as speed up the discovery of new biomarkers^17^.

In this study, we employed egg-based systematic evolution of ligands by exponential enrichment (SELEX), resulting in the identification of a panel of ssDNA aptamers specifically binding to eggs derived from *S. japonicum*. Among these, aptamers LC6 and LC15 exhibited strong binding affinity and specific recognition of *S. japonicum* eggs. Furthermore, tissue imaging results revealed that aptamer LC15 could recognize *S. japonicum* eggs laid in liver tissues with a detection ratio of 80.5%. Collectively, therefore, we obtained novel aptamers specifically recognizing *S. japonicum* eggs, which will facilitate the development of an effective tool for both schistosomiasis diagnosis and drug delivery.

## Results

### Enrichment of DNA library against *S. japonicum eggs*

To obtain aptamers able to recognize Schistosome eggs, an ssDNA oligonucleotide library containing a 45 nt random region flanked by fixed primer regions on the 5′ and 3′ ends was designed and synthesized as an initial selection library. *S. japonicum* eggs were used as the target. *Clonorchis sinensis* (*C. sinensis*) and Schistosoma species are the same flat-shaped trematodes, which can secrete its eggs into the gut of the host and their eggs are passed out with stool. Considering that *C. sinensis* eggs were similar in shaped and size with Schistosome eggs, and can be eligible for a sufficient number by animal experiment *in vitro*, *C. sinensis* eggs was employed as negative control for counter selection.

**Figure 1:**
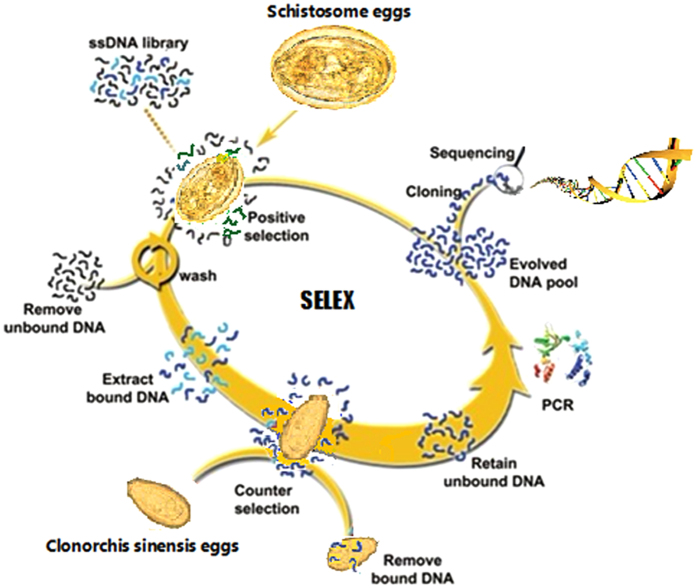
Schematic representation of egg-based aptamer selection. ssDNA library was incubated with target *Schistosoma japonicum* eggs. After washing, the bound DNAs were eluted and then incubated with *Clonorchis sinensis* eggs (negative control) for counter-selection to remove nonspecific sequences. After centrifugation, the supernatant was collected, and DNA was amplified by PCR for next-round selection. When the library was enriched, DNA was sequenced by a high-throughput Illumina sequencing.

The egg-SELEX process is schematically shown in Fig. 1. For the first two selection rounds, we only applied *S. japonicum* eggs for positive selection to enrich ssDNA sequences, to the extent possible, on target cells. From the third round, the ssDNA pool was incubated first with *C. sinensis* eggs to remove nonspecific sequences, and then unbound DNA sequences were collected and further incubated with target *S. japonicum* eggs for positive selection. The ssDNA pool collected after each round of selection was amplified by PCR for next-round selection.

**Figure 2:**
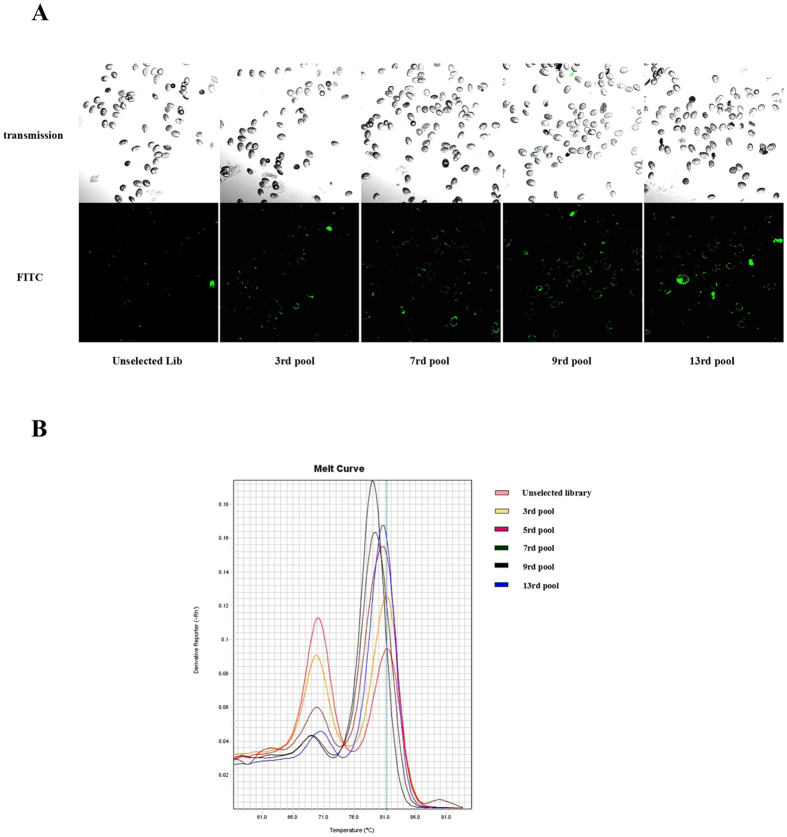
Binding assay of enriched ssDNA pools with *S. japonicum* eggs. (**A**) Confocal microscopy assay of enriched pools binding to *S. japonicum* eggs. (**B**) Melting profiles of aptamer pool from selected individual SELEX rounds.

Since *S. japonicum eggs* are too large for flow cytometry, the enrichment of the aptamer selection process was monitored by confocal imaging using FAM-labeled aptamers. As shown in Fig. 2A, the fluorescence signal on the surface of *S. japonicum* eggs gradually increased with an increasing number of selection rounds, indicating that ssDNA sequences with better binding affinity to *S. japonicum* eggs had been enriched. Furthermore, melting curve analysis based on DNA renaturation/reassociation kinetics (*c*_*0*_*t*) was employed for qualitative estimation of the diversity of aptamer pool, as previously described for monitoring the progress of *in vitro* aptamer selection^18^,^19^. With decreasing diversity of SELEX pool based on increasing enrichment of target-binding ssDNA sequences, the shape and position of the melting curves will change accordingly. As shown in Fig. 2B, the diversity of the pool had already begun to decrease by the third round, whereas aptamer pools in the ninth round showed the greatest enrichment. Therefore, ssDNA pool from the ninth round was submitted to high-throughput Illumina sequencing.

### Identification of aptamer candidates against *S. japonicum* eggs

**Figure 3:**
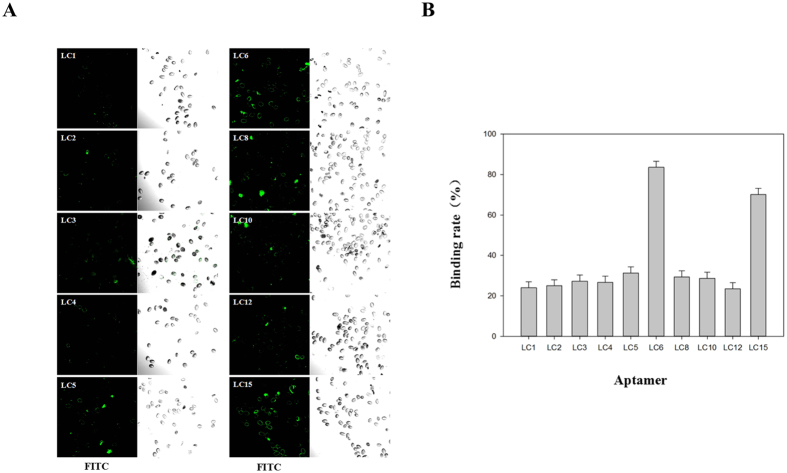
Binding assay of aptamer candidates against *S. japonicum* eggs. (**A**) Confocal microscopy assay of aptamer candidates binding to *S. japonicum* eggs. (**B**) Binding rate of aptamer candidates to *S. japonicum* eggs.

To identify individual aptamer binding to *S. japonicum* eggs, the sequenced aptamer candidates were classified into different groups based on their sequential repeatability, secondary structures and homogeneity. Ten representative sequences from different groups were chosen and synthesized for further characterization. The detailed sequences are listed in Table 1. The binding ability of the selected sequences was evaluated using laser confocal fluorescence microscopy. Two aptamer candidates, LC6 and LC15, showed significant binding to *S. japonicum* eggs (Fig. 3A). The binding rates of LC6 and LC15 were 83.6 and 70.2%, respectively (Fig. 3B).

### Specificity of selected aptamers

**Figure 4:**
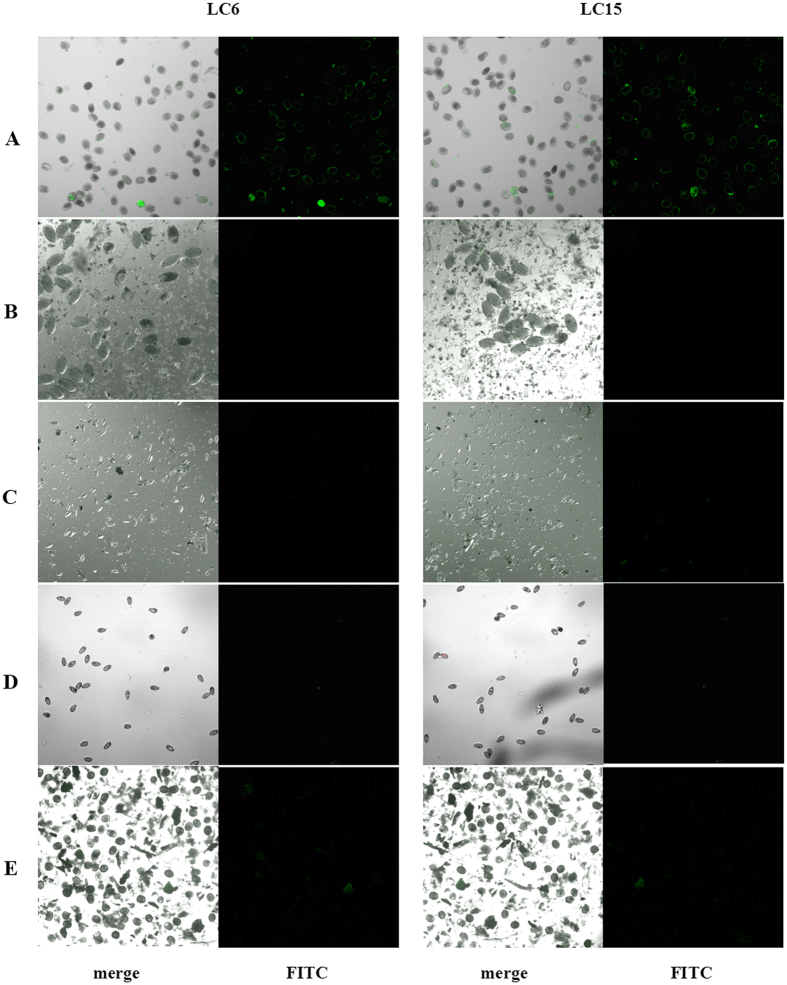
Assay testing the specificity of aptamers LC6 and LC15 to helminth eggs. After aptamers LC6 and LC15 were incubated with *S. japonicum* eggs (**A**), *Fasciolopsis buski* eggs (**B**), *Enterobius* eggs (**C**), *Clonorchis sinensis* eggs (**D**) and *Ascaris* eggs (**E**), respectively, binding ability was assayed using confocal microscopy.

Similar to *S. japonicum* eggs, some intestinal helminth eggs from *Fasciolopsis buski*, *Enterobius* and *Ascaris* were also passed out with stool. They were frequently confused with Schistosome eggs in clinical microscopic examination. Therefore, to investigate the binding specificity of aptamer candidates, these eggs were chosen for specificity evaluation. After FAM-labelled LC6 and LC15 were incubated with *S. japonicum* eggs or other helminth eggs, laser confocal microscopy analyses revealed that the fluorescence signals were only observed on the surface of *S. japonicum* eggs, but not the others, showing that LC6 and LC15 could specifically bind to *S. japonicum* eggs (Fig. 4). This result implied that the cognate cell-surface targets of LC6 and LC15 might only be expressed by *S. japonicum* eggs.

### Imaging of *S. japonicum* eggs in liver tissues

**Figure 5:**
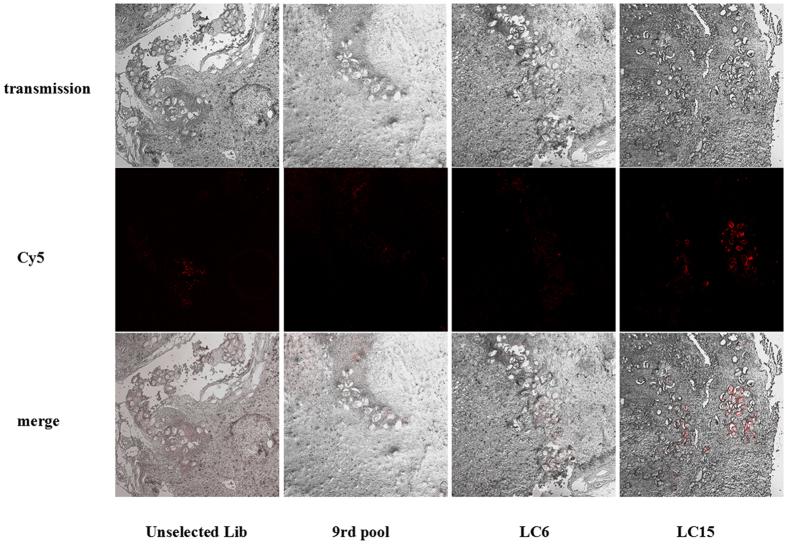
Representative florescence images of rabbit liver tissue sections infected by schistosome stained with Cy5-labeled aptamer LC6, LC15, unselected library and ninth pool.

To identify whether LC6 and LC15 could bind to *S. japonicum* eggs trapped in liver tissues, Cy5-labelled LC6 and LC15 were tested with paraffin-embedded liver tissue sections from infected rabbits. As expected, unselected library and the ninth pool did not bind to *S. japonicum* eggs trapped in liver tissues. However, although both LC6 and LC15 showed significant binding to purified *S. japonicum* eggs *in vitro*, only LC15 could distinguish *S. japonicum* eggs from liver tissues (Fig. 5).

### Stability of aptamer in serum

**Figure 6:**
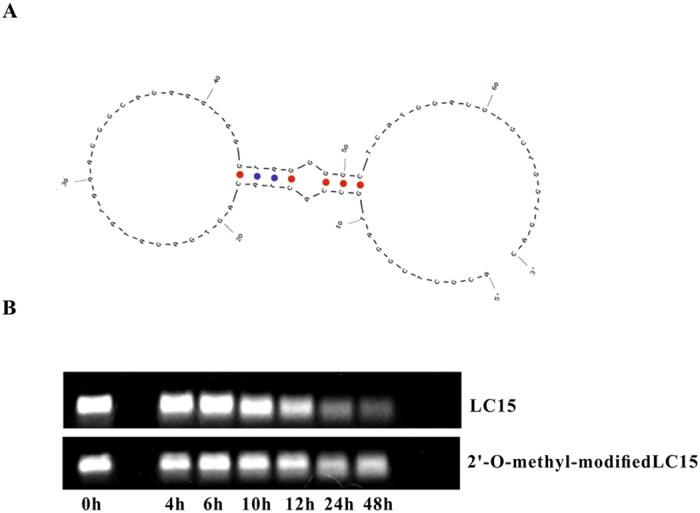
Analysis of aptamer LC-15 stability in serum. (**A**) Secondary structures of aptamer LC15. (**B**) Aptamer LC15 and 2′-O-methyl-modified LC15 were incubated in 1640 medium with 10% FBS for different times.

Aptamer LC15 could bind to *S. japonicum* eggs trapped in liver tissues, thus demonstrating a potential *in vivo* application. Therefore, we further modified the bases of LC15 to enhance its stability in serum. More specifically, the four bases in the 5′ and 3′ termini of LC15 were replaced by 2′-O-methyl oligonucleotides (Fig. 6A). Laser confocal fluorescence microscopy analysis revealed that the 2′-O-methyl substitution did not result in the loss of binding ability of LC15 on *S. japonicum* eggs. Subsequently, cell medium with 10% FBS was incubated with or without 2′- O-methyl oligonucleotide-modified LC15 for the indicated time period. Compared to unmodified LC15, the serum stability of 2′- O-methyl oligonucleotide-modified LC15 was obviously enhanced (Fig. 6B). This result suggests that LC15 modified with 2′-O-methyl oligonucleotides could provide a nuclease-resistant aptamer for further applications.

## Discussion

Aptamers can be generated from a synthetic random library by an *in vitro* iterative selection process called systematic evolution of ligands by exponential enrichment (SELEX). Since the SELEX procedure was established in the 1990s by the independent research groups of Gold and Ellington^8^,^20^,^21^, many aptamers have been generated against various targets, including small organic molecules and metal ions, proteins, cells and tissues. In this study, *S. japonicum* eggs were first employed as targets for aptamer screening. Two specific aptamers against *S. japonicum* eggs, LC6 and LC15, were successfully obtained through an iterative selection process. Although both aptamer LC6 and LC15 showed strong binding ability to purified *S. japonicum* eggs *in vitro*, they exhibited different features when applied to tissue detection. Specifically, only aptamer LC15 could distinguish *S. japonicum* eggs from liver tissues. We inferred that the internal environment in tissues might affect the binding targets of LC6 on the surface of *S. japonicum* eggs, possibly leading to the loss of recognition ability of LC6 on *S. japonicum* eggs trapped in liver tissues.

In this study, the *C. sinensis* eggs were employed for counter selection in the selection process. Aptamer selection by intact biological entities, such as cells and tissue sections, does not require prior knowledge of the complex entities and is not restricted by the properties of targets, providing a new approach for biomarker discovery^22^,^23^. So far, we do not know the biomarkers for *S. japonicum* eggs. However, identification of such biomarkers would greatly improve the diagnosis and treatment of Schistosomiasis.

Currently, the diagnostic gold standard of *Schistosomiasis japonicum* is the detection of eggs with characteristic spines in stool. However, some other helminth eggs, as well as some plant cells, are morphologically similar to *S. japonicum* eggs, making it difficult to distinguish using conventional microscopy. Meanwhile, low-intensity infections reduce the sensitivity of other tests like the Kato-Katz thick smear method. In this study, two aptamers, LC-6 and LC-15, strongly bound to the surface of live *S. japonicum* eggs, not other intestinal helminth eggs. The high affinity and specificity to *S. japonicum* eggs laid in liver tissues imply that selected aptamers hold great promise for accurate diagnosis and targeted therapy, in addition to providing new avenues of research for other diseases like Schistosomiasis.

## Materials and Methods

### Preparation of *Schistosoma japonicum* eggs

All animal protocols were approved by the Animal Ethics Committee of Hunan University and were carried out in accordance with the approved guidelines. Briefly, female rabbits were inoculated with *schistosomal japonicum* cercarials that was sourced from infected Oncomelania hupensis snails collected in Anhui Province, China. After 45 days, the rabbits were sacrificed and their livers viscerated. The livers were shredded and appropriate amount of biological brine containing 1.2% of NaCl was added, the livers were ground quickly into homogeneous slurry with a homogenizer. After removing the residual tissues with a series of sieves (40, 80, 100, 150, 200 mesh/in, respectively), the liquid portion was poured into a conical graduate and allowed to stand to collect the sediment. The sediment was washed with biological brine (1.2% NaCl) for several times until the supernatant was clear. And digestion of liver parenchymal tissues with 0.25% Trypsin-EDTA buffer or 0.1% collagenase B in phosphate buffered saline (PBS). The eggs were incubated in enzyme for 2 h at 25 °C with agitation. For further purification, eggs were centrifuged in Percoll gradients as described by Dalton *et al.*^24^. The collected eggs were preserved in biological brine (1.2% NaCl) at 4 °C to avoid hatching of the eggs.

### Preparation of *Clonorchis sinensis* eggs

Pseudorasbora parva were collected in *Clonorchis sinensis* endemic areas, of which those infected were taken to the lab by muscle tabletting method. Artificial digestive juices were preparated (2 g 1:3000 pepsin and 0.7 mL hydrochloric acid with 100 mL distilled water) according to the proportion of 1:10 for the fish and digestive juices. The liquid was set overnight at 37 °C and filtered by 80 mesh nylon silk screener. The sediment was moved out and the filtrate was processed with water precipitation to collect capsule larva. Sprague dawley (SD) was infected with 50 adolescarias of *Clonorchis sinensis* each by oral injection for stool examination 1 month later. The liver of infected SD was then anatomized to clean the adult worms by sterilized saline water twice in the liver and gallbladder tube through one-time dropper. Each orifice was filled 10 obtained adult worms with with 2 mL 10% calf serum and 1640 medium of amphotericin in an aseptic operation box. Then it was cultivated at 37 °C in a CO_2_ incubator. The culture solution was updated and dead worms were eliminated every 24 h. After that, the culture solution was conducted low speed centrifugal for 3 h. The eggs of *clonorchis sinensis* in the precipitation were obtained. 1.5 mL of the product was then kept in centrifuge tube by using physiological saline at 4 °C. The eggs were collected every 24 h for consecutive 2 weeks.

### Preparation of other *Helminth* eggs

Other *Helminth* eggs such as *Fasciolopsis buski* eggs, *Ascaris* eggs and *Enterobius* eggs were prepared by using nature sedimentation method. Briefly, 20–30 g of stool samples were collected and made suspension with water, which was then filtered through a metal mesh (40–60 mesh metal screener) or 2 to 3-layer wet gauze. Then, let the filtered solution stand for 25 min. Pour the supernatant, fill up clean water every 15–20 min and repeat the process 3 to 4 times until the supernatant was clear. Finally, the supernatant was discharged and stool examination was conducted by microscope.

### DNA library, primers and buffers

The library used in this egg-based cell-SELEX was a 45-nucleotide (nt) randomized region flanked by 20-nt sequences for primer annealing (5′- ACGCTCGGATGCCACTACAG-45N-CTCATGGACGTGCTGGTGAC-3′). For PCR amplification, biotin-labeled reverse primer (5′-biotin-GTCACCAGCACGTCCATGAG-3′) and FAM-labeled forward primer (5′-FAM-ACGCTCGGATGCCACTACAG-3′) were used. All DNA sequences used in the cell-SELEX were purchased from Takara (Dalian, China). Binding buffer was prepared with Dulbecco’s phosphate buffered saline (D-PBS) supplemented with 4.5 g/L of glucose, 0.1 mg/mL of yeast tRNA, 5 mM of MgCl2, and 1 mg/mL of BSA. Washing buffer was prepared with D-PBS supplemented with 4.5 g/L of glucose and 5 mM of MgCl_2_.

### Whole *S. japonicum* egg selection

In this study, *S. japonicum* eggs were used as the target. In total, 20 nmol of DNA library were dissolved in 500 μL of binding buffer. The DNA pool was denatured at 95 °C for 5 min and quickly cooled on ice for 10 min. Along with the above step, we selected about 8000 schistosome eggs. They were washed twice with washing buffer (4.5 g/L glucose and 5 mM MgCl_2_ dissolved in D-PBS) and centrifuged at 1000 r/min for 5 min at 4 °C. The washed eggs were then incubated in a 1.5 mL microcentrifuge tube with the DNA pool and binding buffer at 4 °C in an orbital shaker. After incubation, the eggs were washed to remove unbound DNA sequences. The bound DNA sequences were eluted with 500 μL water by heating at 95 °C for 10 min. The bound sequences were amplified by PCR (8–16 cycles of 30 s at 95 °C, 30 s at 59.4 °C, and 30 s at 72 °C, followed by 5 min at 72 °C), using FAM- and biotin-labelled primers. The double-stranded DNA (dsDNA) was separated from the biotinylated antisense ssDNA by 200 mM sodium hydroxide and streptavidin-coated Sepharose beads (GE Healthcare), desalted and lyophilized for the next round of selection. Starting from the third round, the evolved ssDNA pool was first incubated with about 4000 *Clonorchis sinensis* eggs at 4 °C for 30 min for subtractive selection. The entire selection process was repeated according to the extent of enrichment. Starting from the second round, the evolved ssDNA pool was first incubated with about 8000 *S. japonicum* eggs at 4 °C for 30 min for subtractive selection. The unbound ssDNAs were then removed. For more stringent selection, the number of selection rounds was increased, while decreasing the positive incubation time from 2 h to 1 h. The number of *S. japonicum* eggs was reduced from 8000 to 4000, and the washing times were also extended gradually from 2 to 4 times. At the same time, negative incubation time was gradually increased from 1 h to 2 h. The number of eggs increased from 4000 to 8000. The evolved ssDNA pool generated from the ninth round was PCR-amplified, followed by high-throughput sequencing with Illumina MiSeq (Sangon Biotech Co., Ltd., Shanghai, China).

### Confocal microscopy imaging

After two thousands of eggs were washed with cold washing buffer, the eggs were incubated with FAM-labeled aptamer (200 nM) in 500 μL of binding buffer at 4 °C for 1 h. After washing twice, the eggs were imaged by a FV1000-X81 confocal microscope (Olympus, Japan). The images were analyzed by FV10-ASW Version 3.1.

### Real-time PCR for quantification of target-bound ssDNA

A real-time PCR assay was established to quantify target-bound oligonucleotides from each SELEX cycle. The enriched pool was amplified by PCR using unlabeled primers, and the Applied Biosystems 7500 Fast Real-Time PCR System was used to detect the amplification curve and melting curve of the PCR products. This analysis was performed with 20 μL of reaction mixture containing10 μL KOD SYBR^®^ qPCR Mix, 4 pmol of each primer, 1 × ROX 2 μL, and 100 pM of enriched library. The PCR protocol consisted of 2 min of denaturation, followed by 40 cycles of 10 s at 95 °C, 10 s at 60 °C,annealing temperature, and 30 s at 72 °C for elongation. After amplification, an initial melting curve analysis was performed after the final elongation at 72 °C and then a second remelting analysis after a short reannealing phase. Different reannealing temperatures and times were tested for reMelting Curve Analysis (rMCA). Reannealing was tested for 30 s and 1 min at 60 °C, 70 °C and 75 °C. The melting rates for both melting curve analyses at 95 °C were set at 1 °C s^−1^.

### Immunostaining of Paraffin-Embedded Tissue Sections by LC15-Cy5 and LC6-Cy5

For aptamer staining, tissue sections were deparaffinized three times in xylene and washed with 100% ethanol, followed by 95, 80, 70 and 30% ethanol. For antigen retrieval, tissue sections were boiled in Tris-EDTA buffer (pH 8.0) for 15 min, followed by incubating in binding buffer (PBS with 20% fetal calf serum and 1 mM DNA sodium salt from calf thymus) at room temperature for 1 h. After two washes with washing buffer, sections were incubated with 500nM random sequence and then incubated with 250 nM LC15-Cy5 and LC6-Cy5 for 1 h. The sections were washed three times with washing buffer (1 liter of D-PBS to which were added 4.5 g of glucose and 5 mL of 1 M MgCl_2_), sealed, and observed by fluorescence microscopy.

### Stability of aptamer in serum

100 μM FAM-labeled aptamer or 2′-O-methyl-modified aptamer were incubated in 1640 medium with 10% FBS for different times (0 h, 4 h, 6 h, 10 h, 12 h, 24 h and 48 h) at 37 °C. At the assigned time, samples were flash frozen in a dry ice/ethanol bath and then stored at −80 °C until all samples were harvested. Samples were then thawed on ice and run on 3% agarose gels. Band density was assayed by a molecular imager (Bio-Rad).

## Additional Information

**How to cite this article**: Long, Y. *et al.* Screening and identification of DNA aptamers toward *Schistosoma japonicum* eggs via SELEX. *Sci. Rep.* **6**, 24986; doi: 10.1038/srep24986 (2016).

## Figures and Tables

**Table 1:** Sequences of aptamer candidates.

Clone ID	Sequence of random region (5′ to 3′)
LC1	GTAATGAGAAGTAAGTATAGGTATTGAGAGTATGAGGG
LC2	ACGCTCGGATGCCACTACAGTAGTAGGAG
LC3	TGAGTAAAGATTCGCGAGTTAGAGATAGAAAATGAGT
LC4	GGTAGAGTAGGTAATTGATAAGGCGTTGAGTAAGTAGGTAT
LC5	CAGTCGGAGTAGAGTTATAGTATGGTGTTTAGAAATTGAT
LC6	TGATAGAATGGTAGTTGAGTAGTTTGTGTATATGTGGGGC
LC8	CGGTATTTGTAAGAGTAGGTAATGTGTAGAGTTAGTAGCC
LC10	CTCGGAATGATGAGGGTAAATATTGTATGGTGACGATGGA
LC12	TTAGAGAGATAGTAGATAGATAATTAAGGTAAT
LC15	TGAGATATAAAGGGCAGAAATAAGTAGGGG
